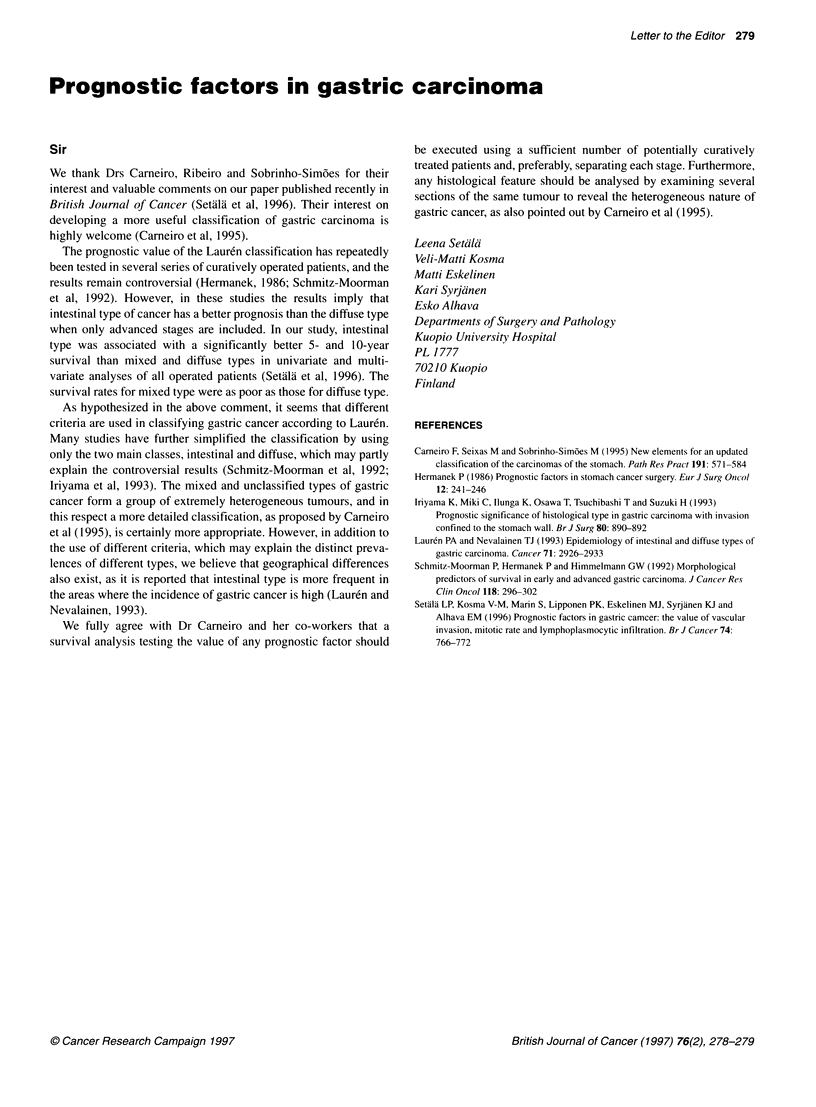# Prognostic factors in gastric carcinoma - reply

**Published:** 1997

**Authors:** Leena Setälä, Veli-Matti Kosma, Matti Eskelinen, Kari Syrjänen, Esko Alhava


### Prognostic factors in gastric carcinoma

### Sir

We thank Drs Carneiro, Ribeiro and Sobrinho-Simões for their interest and valuable comments on our paper published recently in *British Journal of Cancer* (Setälä et al, 1996). Their interest on developing a more useful classification of gastric carcinoma is highly welcome (Carneiro et al, 1995).

The prognostic value of the Laurén classification has repeatedly been tested in several series of curatively operated patients, and the results remain controversial (Hermanek, 1986; Schmitz-Moorman et al, 1992). However, in these studies the results imply that intestinal type of cancer has a better prognosis than the diffuse type when only advanced stages are included. In our study, intestinal type was associated with a significantly better 5- and 10-year survival than mixed and diffuse types in univariate and multivariate analyses of all operated patients (Setälä et al, 1996). The survival rates for mixed type were as poor as those for diffuse type.

As hypothesized in the above comment, it seems that different criteria are used in classifying gastric cancer according to Laurén. Many studies have further simplified the classification by using only the two main classes, intestinal and diffuse, which may partly explain the controversial results (Schmitz-Moorman et al, 1992; Iriyama et al, 1993). The mixed and unclassified types of gastric cancer form a group of extremely heterogeneous tumours, and in this respect a more detailed classification, as proposed by Carneiro et al (1995), is certainly more appropriate. However, in addition to the use of different criteria, which may explain the distinct prevalences of different types, we believe that geographical differences also exist, as it is reported that intestinal type is more frequent in the areas where the incidence of gastric cancer is high (Laurén and Nevalainen, 1993).

We fully agree with Dr Carneiro and her co-workers that a survival analysis testing the value of any prognostic factor should be executed using a sufficient number of potentially curatively treated patients and, preferably, separating each stage. Furthermore, any histological feature should be analysed by examining several sections of the same tumour to reveal the heterogeneous nature of gastric cancer, as also pointed out by Carneiro et al (1995).

*Leena Setälä*
*Veli-Matti Kosma*
*Matti Eskelinen*
*Kari Syrjänen*
*Esko Alhava*
*Departments of Surgery and Pathology*
*Kuopio University Hospital*
*PL 1777*
*70210 Kuopio*
*Finland*

### REFERENCES

Carneiro F, Seixas M and Sobrinho-Simões M (1995) New elements for an updated classification of the carcinomas of the stomach. *Path Res Pract* **191**: 571–584

Hermanek P (1986) Prognostic factors in stomach cancer surgery. *Eur J Surg Oncol* **12**: 241–246

Iriyama K, Miki C, Ilunga K, Osawa T, Tsuchibashi T and Suzuki H (1993) Prognostic significance of histological type in gastric carcinoma with invasion confined to the stomach wall. *Br J Surg* **80**: 890–892

Laurén PA and Nevalainen TJ (1993) Epidemiology of intestinal and diffuse types of gastric carcinoma. *Cancer* **71**: 2926–2933

Schmitz-Moorman P, Hermanek P and Himmelmann GW (1992) Morphological predictors of survival in early and advanced gastric carcinoma. *J Cancer Res Clin Oncol* **118**: 296–302

Setälä LP, Kosma V-M, Marin S, Lipponen PK, Eskelinen MJ, Syrjänen KJ and Alhava EM (1996) Prognostic factors in gastric camcer: the value of vascular invasion, mitotic rate and lymphoplasmocytic infiltration. *Br J Cancer* **74**: 766–772

© Cancer Research Campaign 1997